# Foreword to the Special Issue: HCM in 2018

**DOI:** 10.21542/gcsp.2018.37

**Published:** 2018-08-12

**Authors:** Perry Elliott

**Affiliations:** 1 Institute of Cardiovascular Sciences, University College London, London, UK; 2 St Bartholomew’s Centre for Inherited Cardiovascular Disease, St Bartholomew’s Hospital, West Smithfield, London, UK

The first description of hypertrophic cardiomyopathy (HCM) can be attributed to any number of great anatomists and physicians working from the 17th century onwards, but the modern era is often said to have begun with Donald Teare’s landmark paper of 1958^1^ that spurred a period of intense clinical investigation that has continued unabated to the present day. The fruits of this collective endeavour include an understanding of the genetic architecture of the disease, an appreciation of its complex pathophysiology, and much progress in clinical management. However, many challenges remain, particularly with respect to disease prevention and the management of progressive heart failure.

HCM differs in several important aspects from other conditions associated with left ventricular failure and cardiac hypertrophy. From a clinical perspective it has a well-known association with sudden cardiac death but it also represents an important prototype for diastolic heart failure. The natural history of heart failure in HCM is notably longer than that of ischaemic heart disease with symptom evolution and remodelling occurring over decades. In about one third of patients the presence of intra-cavity or left ventricular outflow tract obstruction provides an additional haemodynamic burden and endothelial and microvascular dysfunction are present from an early stage and contribute to the genesis of symptoms and disease progression. The prognosis of HCM is also highly variable with sudden death the major cause of mortality in younger patients and heart failure and stroke the major causes of death in older individuals. Earlier diagnosis within families has also led to a growing number of healthy carriers of pathogenic mutations that offer opportunities for early intervention to prevent disease.

In this special issue of *Global Cardiology Science and Practice*, we present a unique series of papers that review the aetiology, clinical presentation and management of HCM from childhood to old age. The series reveals how the simple notion of HCM as a disease defined by hypertrophy unexplained by loading conditions, encompasses a remarkably complex spectrum of genetic and acquired disorders. Sudden death prevention with implantable cardioverter defibrillators and the management of left ventricular outflow tract obstruction are comprehensively reviewed, but attention is also given to the management of rare but important phenocopies. The nuances of management in particular sub-phenotypes are also discussed and illustrate the need for systematic and expert evaluation of this and other complex heart muscle disorders.

For many decades, the diagnosis and management of HCM has run in parallel with technological developments that permit more precise and timely diagnosis but not necessarily greater insight into pathogenesis or novel therapies. In 2018, however, there are signs that a new and exciting chapter in the story of HCM and other cardiomyopathies has begun with the transition of molecular genetics into the clinic and a growing number of randomised clinical trials designed to ameliorate and perhaps even prevent disease. It is our sincere hope that this expert series will spur readers to learn more about this fascinating group of diseases and to contribute to this landscape of therapeutic opportunity.
